# Hierarchical processing in Balint’s syndrome: a failure of flexible top-down attention

**DOI:** 10.3389/fnhum.2014.00113

**Published:** 2014-02-27

**Authors:** Carmel Mevorach, Lilach Shalev, Robin J. Green, Magda Chechlacz, M. Jane Riddoch, Glyn W. Humphreys

**Affiliations:** ^1^Behavioural Brain Sciences Centre, School of Psychology, University of BirminghamBirmingham, UK; ^2^The Constantiner School of Education, Tel-Aviv UniversityTel Aviv, Israel; ^3^Sagol School of Neuroscience, Tel-Aviv UniversityTel Aviv, Israel; ^4^Department of Experimental Psychology, University of OxfordOxford, UK

**Keywords:** balint’ s syndrome, flexible attention, salience-based selection, disengagement, global processing, local processing

## Abstract

Patients with Balint’ s syndrome are typically impaired at perceiving multiple objects simultaneously, and at evaluating the relationship between multiple objects in a scene (simultanagnosia). These deficits may not only be observed in complex scenes, but also when local elements of individual objects must be integrated into a perceptual global whole. Thus, unlike normal observers, patients with simultanagnosia typically show a bias towards the local forms, even to the extent that they cannot identify the global stimuli. However, we have previously shown that global processing is still attainable in Balint patients in certain scenarios (e.g., when local elements are unfamiliar). This suggests that in addition to a possible perceptual deficit that favors the local elements in these patients, impaired attentional control may be at the core of their unique performance. To test this hypothesis we manipulated the perceptual saliency of the local and global elements in a compound letter task so that it included global-more-salient or local-more-salient displays. We show that a Balint patient was able to accurately identify both global and local targets as long as they were the salient aspect of the compound letter. However, substantial impairment was evident when either the global or local elements were the less salient aspect of the compound letter. We conclude that in Balint’ s syndrome there is a failure of flexible top-down attention both in biasing attention away from salient irrelevant aspects of the display (salience-based-selection) and in impaired disengagement from irrelevant but salient items once they have been selected.

## INTRODUCTION

Balint’s syndrome is a rare neurological disorder associated with bilateral parieto-occipital damage (Balint, 1909). The syndrome typically consists of disturbed organization of eye movements (ocular apraxia), inaccurate reach responses to objects under visual guidance (optic ataxia), impairments of spatial orienting and localization, and impaired ability to detect and identify more than one object or one of its local features at a time (simultanagnosia; Balint, 1909; Rafal, 1996; Rizzo and Vecera, 2002; Karnath and Zihl, 2003). The term simultanagnosia here refers to severe difficulty in interpreting complex, multi-object scenes (such as the Boston Cookie Thief picture), and poor ability to perceive two simultaneously presented objects relative to the presentation of single objects (Kinsbourne and Warrington, 1962; Humphrey et al., 1994; Shalev and Humphreys, 2002). Thus, such deficits are observed not only in complex scenes, but also when separate components are required to be integrated to a single object.

The process of integrating parts into wholes has been examined most extensively in tasks where the patients are asked to respond to the local or global levels of compound shapes, where the global form is derived from the configuration of the multiple local elements (Navon, 1977). It has been shown previously that patients with simultanagnosia demonstrate a bias towards the local forms in such tasks, a bias that in some cases causes a complete failure to perceive the global aspect of the compound item (Humphrey et al., 1994; Karnath et al., 2000a; Jackson et al., 2004; Shalev et al., 2005; Huberle and Karnath, 2006).

One explanation that has been proposed for the deficient global perception in Balint’s syndrome is a narrow and restricted window of attention (Thaiss and De Bleser, 1992; Shalev and Humphreys, 2002; Michel and Henaff, 2004; Dalrymple et al., 2007, 2011, 2013). While the perception of local parts may still operate with a narrow attentional window, global object identification typically requires a distributed spread of attention, which the patients cannot achieve. However, it should be noted that such an explanation cannot provide a full account for several of the findings that have been reported with simultanagnosic patients. One example is illusory conjunctions of color and form which reflect the processing of features of more than one object (Friedman-Hill et al., 1995); others include the ability of simultanagnosics to statistically average across stimuli (Demeyere et al., 2008), to estimate magnitudes (Demeyere and Humphreys, 2007), to perceive one spatial area when elements group but a reduced area when elements segment apart (Luria, 1959; Humphreys and Riddoch, 1993; Gilchrist et al., 1996) and to show implicit processing of global shape (e.g., interference in responding to the local shape when the global shape is incongruent; Karnath et al., 2000b; Jackson et al., 2004; Shalev et al., 2005). Moreover, Balint’s patients can also identify large forms, matched in size to the global compounds they fail to perceive (Shalev et al., 2005). In such cases the impaired global perception of Balint’s patients cannot be the result of a mere inability to spread attention across a wide area. Shalev et al. (2005) additionally showed that attention could be precued by the prior identification of a large solid figure, so that global compound stimuli presented shortly afterwards could also be identified successfully. Thus there is not necessarily a limit on whether attention can be distributed across a wide spatial area, though distributed attention may be difficult to sustain. Consistent with the latter argument, Shalev et al. (2005) found that the perception of global compound stimuli decreased as the time interval between the initial large letter and the compound shape increased.

In an attempt to elucidate key factors determining the spread of attention in these patients, Huberle and Karnath (2006) manipulated the distances between the local letters in compound forms. They found that performance systematically improved as the inter-element distances decreased, keeping constant the global size of the letters (see also Dalrymple et al., 2007; Huberle and Karnath, 2010; Montoro et al., 2011). Reduced inter-element distances presumably promote grouping and the spread of attention across the grouped elements. Familiarity is also a contributory factor. Coslett and Saffran (1991) reported a simultanagnosic patient who named words but not non-words although the spatial characteristics of both words and non-words were the same (see also Baylis et al., 1994; Kumada and Humphreys, 2001). These data suggested that letters in words are grouped so that the word is processed as a single perceptual object whereas letters in non-words are coded as distinct objects. The converse effect, of familiarity disrupting performance, can also occur when local rather than global forms are familiar. Shalev et al. (2007) demonstrated that their simultanagnosic patient perceived the global shape of a compound letter as long as its local elements were unfamiliar; however, after the patient was trained to identify the local (previously unfamiliar) elements, it became difficult to perceive global forms containing the now-familiar local elements.

These various manipulations cannot be boiled down to a single perceptual factor being responsible for simultanagnosia (e.g., differential sensitivity to set spatial frequencies; Huberle and Karnath, 2006); nevertheless it can be argued that the effects represent a variety of manipulations all of which may have an impact on the relative saliency of the local and global levels of stimuli (Shalev et al., 2007). When the local elements have high saliency (and are more salient than the global form), then patients with simultanagnosia will demonstrate “local capture” and only identify the local items. In contrast, when the global configuration is more salient the patients can exhibit global capture (Dalrymple et al., 2007; Montoro et al., 2011). That is, as with normal participants (e.g., Mevorach et al., 2006b, 2010), stimulus characteristics can bias both a narrow or a wide attention window, but once attention is captured at one level, patients with Balint’s syndrome find it difficult to flexibly re-allocate attention to other levels. This reduced flexibility in selective attention is additional to any de-fault bias towards a restricted (local) attentional field. In the present study we directly tested the above explanation in a patient (JM) with Balint’s syndrome.

## THE PRESENT STUDY

In the present study we used a variant of the “classical” global-local identification task with compound letters, as introduced by Mevorach et al. (2006b, 2010). In this variant, the relative saliency of the local/global levels of the compound stimuli is manipulated orthogonally to the level required for target identification (e.g., the target might be at the local level and the global level is rendered salient or vice versa). Our prior work with both healthy participants (e.g., Mevorach et al., 2006b, 2009, 2010) and patients with neuropsychological disorders (Mevorach et al., 2006a), has implicated the left posterior parietal cortex (PPC) in conditions where the distractor is more salient than the target – a finding that arises irrespective of the target level (i.e., the left PPC is critical both when the global level is salient and the task requires identification of a local target, and when the local level is salient and there is a global target). For example, patients with left PPC damage show impairment in identifying both global and local targets which are less salient than the distractor level (Mevorach et al., 2006a).

If the functional deficit in Balint’s syndrome includes diminished flexibility in controlling attentional selection, but if sensitivity to the relative saliency of different levels of form remains, then we should be able to manipulate whether the local or global level of a stimulus is reported by systematically varying the relative saliency of each level. Critically, once a level is selected the patient should be poor at selecting the other level. We tested this here using the manipulations of saliency introduced by Mevorach et al. (2006b, 2010).

## MATERIALS AND METHODS

### CASE REPORT

#### JM

**FIGURE 1:**
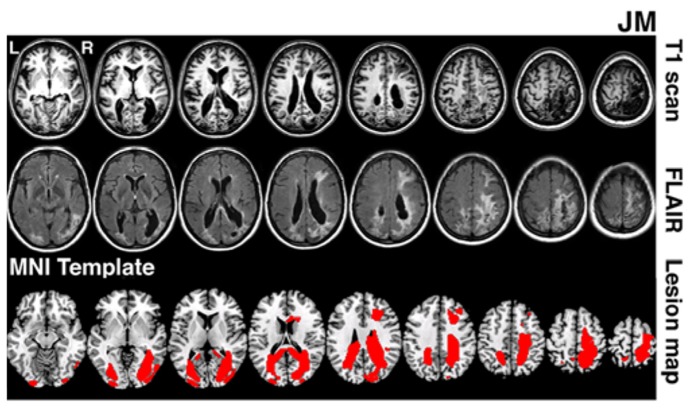
**Lesion reconstruction for patient JM.** The figure depicts T1 and T2 FLAIR structural scans (3T Philips Achieva MRI system with 8-channel phased array SENSE head coil). The bottom panel represents patient’s lesion map reconstructed based on outlier detection method combined with fuzzy clustering algorithm (see Seghier et al., 2008; Chechlacz et al., 2012). The lesion map is presented as an overlay on a standard T1 multi-slice template in MRIcron (Chris Rorden, Georgia Tech, Atlanta, GA, USA). L = Left and R = Right hemisphere.

JM was 45 years old and a housewife at the time of testing. 4 years prior to testing she suffered a bilateral stroke while giving birth. This resulted in bilateral lesions in occipital and parietal cortices extending in the right hemisphere into frontal cortex (lesion volume 141.2 cubic centimetres). MRI scans (T1 and FLAIR) are shown in **Figure 1**. Following the stroke, JM had no major motor weakness but presented with symptoms characteristic of Balint’s syndrome, she has optic ataxia, with inaccurate visually guided reaching to objects, especially in peripheral vision. She showed signs of ocular apraxia, with a poor ability to make saccades to peripheral signals. She had simultanagnosia. She found it very difficult to identify the events in visual scenes, reporting only on the presence of a woman washing dishes in the Boston cookie theft picture. In a test of visual extinction she required over 2 s to be able to identify two letters though she was able to identify single letters presented in either her left or right field for only 200 ms. These two deficits, in interpreting complex scenes and in identifying more than one object at a time, are key defining symptoms of simultanagnosia (Kinsbourne and Warrington, 1962). There was no evidence for spatial bias in JM’s performance – and she identified about half of the letters in the right field and half in the left field under the extinction conditions (above). Also she only canceled lines down the center of the page in a cancelation task. JM’s single word reading was good (12/12 for both regular and irregular words matched for length and frequency) but text was extremely difficult (even reading single sentences was not possible). Her identification of single objects was relatively spared (13/15 on naming items from the BCoS battery; Humphreys et al., 2012). Verbal long- and short-term memory was good (forward digit span = 6; backwards digit span = 4; story recall from the BCoS was within normal limits, 11/15).

### CONTROL PARTICIPANTS

**FIGURE 2:**
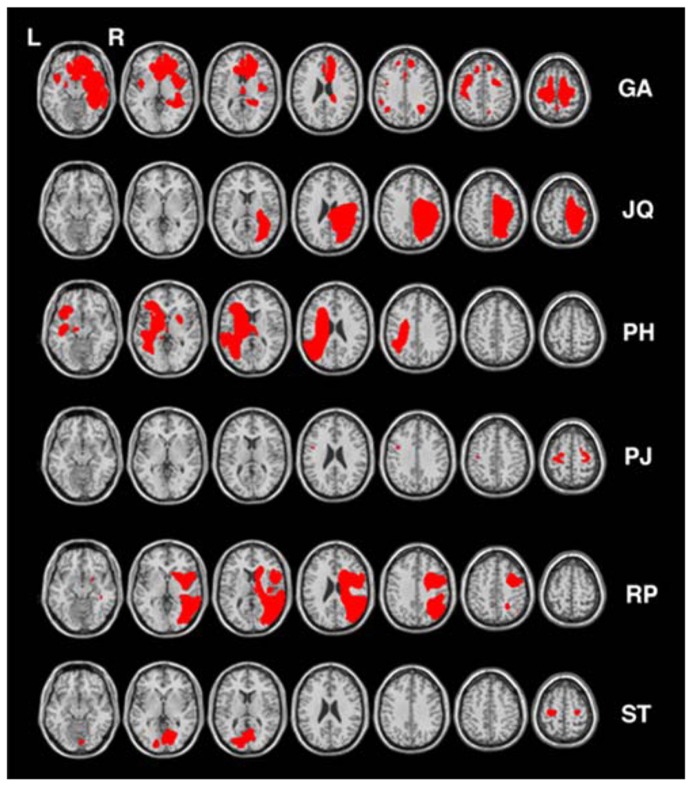
**Lesion reconstruction for all control patients.** Each patient’s lesion map was reconstructed based on outlier detection method combined with fuzzy clustering algorithm (see Seghier et al., 2008; Chechlacz et al., 2012). All lesion maps are presented as an overlay on a standard T1 multi-slice template in MRIcron (Chris Rorden, Georgia Tech, Atlanta, GA, USA). L = Left and R = Right hemisphere.

Six other neurologically impaired patients, all male, were tested (see **Table 1** for their clinical details). The patients were selected to represent an age matched patient control group for JM and to include a range of neuropsychological problems including neglect (patient RP), dysexecutive function (GA, JQ), extinction (PH) and visual field loss (ST). Using a patient control group here can ascertain that any difficulty observed in JM is not attributed to a general non-specific reduced capacity that often accompanies brain lesions or a specific spatial deficit such as unilateral neglect, extinction and field loss. The neuropsychological symptoms of the patients are listed in **Table 1**. Prior to participating in the study the patients were clinically assessed using the BCoS battery (Humphreys et al., 2012) and T1 structural MRI scans were acquired (see **Figure 2**). The neuropsychological symptoms described in **Table 1** reflect instances where performance fell 3 SD’s > mean for that participant on tests from the BCoS for memory, executive function, picture naming, extinction and visual field loss.

**Table 1:** Control patients with their associated ages, gender and lesion information.

Patient initials	Age at test (years)	Neuropsychological deficit	Type of brain injury	Time since injury (years)	Lesion side, location	Lesion volume (cc)
GA	56	Amnesia, dysexecutive symtpoms	Other^*^	16	B, temporal, frontal	154.0
JQ	57	Dysexecutive symptoms	Stroke	2	R, fronto-parietal	122.9
PH	38	Aphasia, dyslexia, right extinction	Stroke	12	L, frontal	108.7
PJ	42	Aphasia	Other^@^	2	No lesion on scan	
RP	56	Left neglect, extinction	Stroke	6	R temporo-parietal	119.7
ST	54	Visual field defect	Stroke	3	B, occipital	10.5

* Herpes simplex encephalitis; ^@^unspecified vasculitis; cc, cubic centimetres; B, bilateral; L, left; R, right.

### STIMULI

**FIGURE 3:**
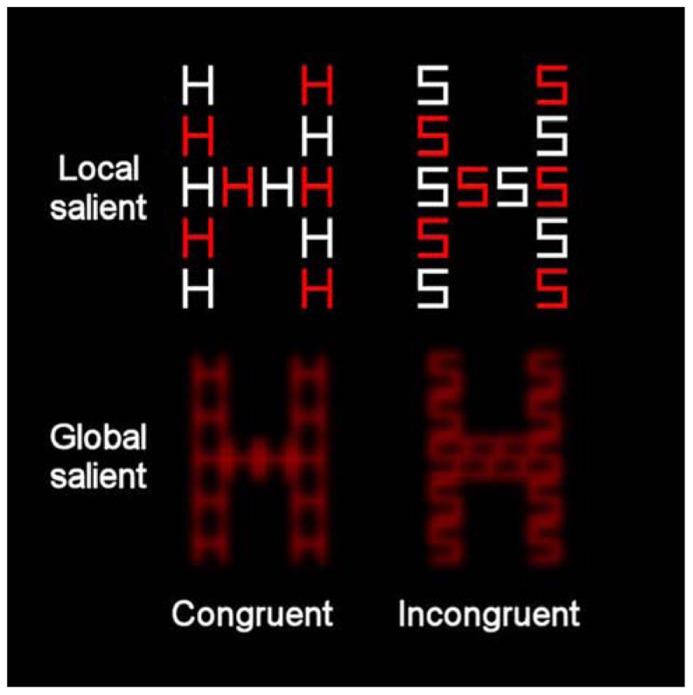
**Example of the compound letters that were used in the present study incorporating global-more-salient and local-more-salient displays.** The levels of each stimulus could be congruent (where local and global letters matched) or incongruent (where local and global letters differed).

The stimuli were presented on a 17-in. monitor (1024 × 768 pixels) of a laptop. The viewing distance was approximately 60 cm so that each centimeter on the screen represented 0.968 of visual angle. All the stimuli appeared against a black background. Two sets of displays were used to represent high global saliency and high local saliency. For the condition with relatively high local saliency, the compound stimuli were created from orthogonal combinations of the letters H and S. Each compound contained both red and white local letters (see **Figure 3**). Each local letter subtended 1.348 × 1.068 of visual angle (in width and height, respectively) and the global letter subtended 8.268 × 5.388 of visual angle (in width and height, respectively). The inter-element distance was 0.388 of visual angle. In the condition with relatively high global saliency, the compound letters were again composed of the letters H and S, which were combined orthogonally at the local and global levels. All the local letters were red. Each local letter subtended 1.348 × 1.068 of visual angle (in width and height, respectively) and the global letter subtended 5.668 × 4.518 of visual angle (in width and height, respectively). The distance between local elements was 0.0968 of visual angle. These letters underwent a blur procedure in Paint Shop Pro 7.0 with factor = 7. The compound letters appeared at the center of the screen. A white asterisk (0.578), also presented at the center of the screen, served as fixation.

### EXPERIMENTAL PROCEDURE

Prior to the experiment JM was shown individual large block letters, matched in size to the global letters in the experiment and she was required to name the letters. She scored perfectly. We also showed her the blurred stimuli and asked her to name single letters when the remaining parts of the global shape were covered up. She was able to do this (10/10). On the subsequent blocks of 32 trials, JM was presented with compound (local-global) letters. She was instructed that the global letter was the same size as the large block letters she had just seen, and that the local letter was the same size as the individual small letters she had identified. She was then required to verbally identify the global or the local elements of the compound letter (while ignoring the identity of the letters on the other irrelevant level). In each block half of the trials consisted of matching global and local elements (congruent trials), and on the other half different global and local elements comprised the compound figure (incongruent trials). Each trial began with a fixation asterisk presented for 500 ms. Following a 200-ms interval, the compound letter appeared for 150 ms. JM was then required to make a speeded response to the identity of the letter on the target level (H or S) by verbally stating its identity (“H” or “S”) following which the experimenter pressed one of two keyboard keys (“m” and “k,” for H and S, respectively). Following the key press, the next trial began. Each run of the task included four blocks (two with “global” targets and two with “local” targets; 128 trials). A written instruction (“global task” or “local task”) appeared at the center of the screen 2 s prior to the beginning of each block at which time the experimenter also indicated the instruction to the patient. The first two blocks and the last two blocks of each run had the same display – either global level being more salient or local level being more salient (the order was counterbalanced across runs). Thus, four different types of blocks were used: Global target with global-salient display, Local target with global-salient display, Global target with local-salient display and Local target with local-salient display. JM completed two runs of the task. The same procedure was administered to the six control patients.

In order to manipulate saliency at both global and local levels we coded the saliency factor according to whether the target level was more salient (Target-salient) or the distractor level was more salient (Distractor-salient). Thus, the global block with global-salient displays was considered a global task in the Target-salient condition while the global block with locally salient displays was labeled a global task in the Distractor-salient condition. Similarly, for the local task, the block with locally salient displays was considered the Target-salient condition and the block with globally salient displays was considered the Distractor-salient condition (see **Table 2**).

**Table 2:** Assignment of the four blocks to the level and saliency conditions.

	Global		Local	
	Target salient	Distractor salient	Target salient	Distractor salient
Target level	Global	Global	Local	Local
Display type	Global-salient	Local-salient	Local-salient	Global-salient

## RESULTS

**FIGURE 4:**
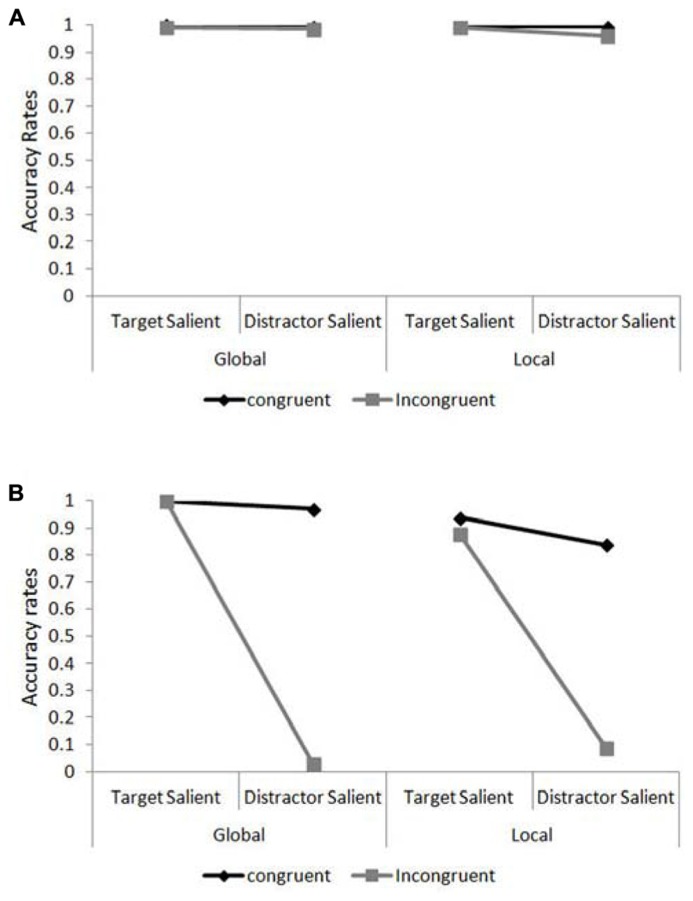
**Accuracy rates in the global/local task as a function of target level and target/distractor saliency.** **(A)** Accuracy rates for the control patients in the compound letter task. **(B)** Accuracy rates for patient JM in the compound letter task.

Accuracy data for JM and the control patients were analyzed using Chi square and Fisher Exact Probability Tests. The percentage of correct responses in the different experimental conditions are presented in **Figures 4A,B** for the control participants and JM, respectively. We asked whether JM was impaired compared to the other patients when she was required to ignore the salient irrelevant level of the compound letter stimuli. For this we calculated the congruency effect (the accuracy for congruent trials minus that for incongruent trials) when the target level had high relative saliency and when the distractor level had high relative saliency (across level of processing). JM’s congruency effect in the target-salient condition was small (62/64; 96.9% correct responses in congruent trials vs. 60/64; 93.8% correct responses in incongruent trials) and similar in magnitude to the control patients (383/384; 99.7% correct responses in congruent trials vs. 380/384; 99.0% in incongruent trials; χ^2^_(1)_ = 0.002, *p* = 0.89). However, in the distractor-salient condition JM was essentially unable to ignore the identity of the salient distractor (she made 58/64; 90.6% correct responses to congruent trials vs. only 4/64; 6.3% correct responses to incongruent trials) in dramatic contrast to the control patients, who showed a modest congruency effect (they made 382/384; 99.5% correct responses to congruent trials vs. 373/384; 97.1% correct responses to incongruent trials; χ^2^_(1)_ = 42.5, *p* <0.0001). This striking inability to report a target on a non-salient level when the distractor level was salient and incongruent was not associated with a particular level of the stimulus. JM showed greatly increased congruency effects, compared with the control patients, for both local and global non-salient targets. For the local task JM responded correctly to 27/32 (84.4%) congruent trials vs. 3/32 (9.4%) incongruent ones; in contrast the controls answered correctly to 191/192 (99.5%) congruent trials vs. 184/192 (95.8%) incongruent ones (χ^2^_(1)_ = 17.1, *p* <0.0001). In the global task JM responded correctly to 31/32 (96.97%) congruent trials vs. 1/32 (3.1%) incongruent one whereas the control participants answered correctly to 191/192 (99.5%) congruent trials vs. 189/192 (98.4%) incongruent ones; Fisher Exact Probability Test, *p* <0.0001. Importantly, for JM the magnitude of the congruency effects in the global and local conditions was similar (for local targets – 27/32 vs. 3/32 and for global targets – 31/32 vs. 1/32 for congruent and incongruent trials, respectively; Fisher Exact Probability Test, *p* = 0.282). It should also be noted that JM (and the control patients) were required to provide a response in each and every trial (there were no “miss” or “pass” trials) and her extremely low accuracy in incongruent trials are therefore attributed to responding to the letter on the distractor level.

It is also evident that local identification for JM was particularly hard under conditions of distractor saliency, where even her performance in congruent trials was relatively poor (27 correct responses out of 32 trials [84.4%] compared with 191/192 [99.5%] of the controls; Fisher Exact Probability Test, *p* <0.0003). JM’s difficulty in identifying the local elements was also evident in her performance under target-salient conditions where her accuracy was overall lower than in the global task (58/64 [90.1%] vs. 64/64 [100%] for local and global identification, respectively; Fisher Exact Probability Test, *p* = 0.014) or that of the controls (58/64 [90.1%] vs. 381/384 [99.2%]; Fisher Exact Probability Test, *p* <0.0004).

## DISCUSSION

Simultanagnosia within the context of Balint’s syndrome has been previously associated with a bias towards processing local items at the expense of global processing (Thaiss and De Bleser, 1992; Shalev and Humphreys, 2002; Michel and Henaff, 2004; Dalrymple et al., 2007, 2011, 2013). However, evidence for at least some aspects of global shape processing in the syndrome (e.g., interference from incongruent global stimuli; statistical averaging; magnitude estimate; Shalev et al., 2005; Demeyere and Humphreys, 2007; Demeyere et al., 2008) indicates that global processing can still operate to some degree and that a constricted attention window cannot be the sole underlying reason for the problem. Here we manipulated local and global forms so that either the local or the global shapes were salient (see Mevorach et al., 2006b, for prior evidence with normal participants). We showed that there was capture of attention by either the local or the global shape, dependent on their relative salience. In each case, our patient JM was typically unaware of the non-selected level and reported only the salient stimulus. The results we report are similar to some prior data where the representation of the global form has been enhanced by using closely aligned local elements (Huberle and Karnath, 2006; Montoro et al., 2011) or shapes constructed to make the global forms salient (Dalrymple et al., 2007). The data provide clear evidence that simultanagnosics do process global form and that their attention can be locked to that level of representation when the global form is high in saliency and the local relatively low in saliency.

In his original report, Balint discussed the inability of his patient to make saccades to stimuli as “psychic paralysis of gaze” (we earlier termed this ocular apraxia). The present results suggest that this “paralysis” is not confined to gaze (overt attention) but affects even covert attention. In the present task, when JM attended to a salient global form she did not need to shift her gaze in order to subsequently attend to a centrally positioned local form (in a local identification task). Her failure to identify the local form then is not a paralysis of gaze but rather a paralysis of attention; she was unable to shift attention from the global to the local level (or vice versa).

One possible alternative explanation for JM’s performance in this study is that our manipulation of perceptual saliency created conditions that are perceptually rather than attentionally difficult for her. For instance, blurring the local elements in the global-salient displays might have created local elements that JM was simply unable to identify, regardless of the presence of the global information. However, we note that JM was able to identify a single blurred local letter when the remaining local letters were covered (she was also able to identify single blocked letters, matched for size to the global stimuli; see Methods). In addition, if JM was simply unable to identify local elements under these conditions we would have expected her performance to be at chance level (indicating her inability to identify the stimuli). However, JM’s performance was considerably below chance and thus reflected identification of the irrelevant (but salient) level. This in itself suggests that JM’s attention was allocated to the irrelevant (but salient) aspect of the compound letter and that she was simply unable to ignore that information (even if no other information was available for her) and to disengage from it. Poor perception of the local letters, but good disengagement from the global form, should generate chance levels of identification for the local stimuli. In contrast, JM performed worse than chance.

In a now classic study, Posner et al. (1984) first documented problems in the disengagement of attention associated with unilateral lesions to the PPC – patients were poor at shifting attention to the contralesional side if attention was earlier cued to the ipsilesional side. Posner et al. argued that a critical function of the PPC was to disengage attention from a given spatial region. This result has been confirmed in subsequent brain imaging studies (see Corbetta and Shulman, 2002, for one review), where it has been argued that the PPC (and in particular the right temporo-parietal junction, rTPJ) acts to detect new events and through this, to trigger the attentional disengagement process (the attentional disengagement account of PPC function). Our data concur with the proposal that the PPC is critical for the disengagement of visual attention – though here the problem is not manifest in poor spatial disengagement (local forms would fall within a spatial window of attention when the global form is selected) but in poor disengagement from one level of form to another. This suggests that the PPC may subserve a number of different forms of attentional disengagement. In addition, our data do not fit with the account of one region of the PPC, the rTPJ, proposed by Corbetta and Shulman (2002). These authors argue that the right TPJ acts as a “circuit breaker” for attention, disengaging attention from its current focus on the occurrence of an unexpected, salient stimulus. Note that, in our study, disengagement of attention from a high to a low saliency stimulus is not triggered by the occurrence of an unexpected event, since the low salient aspects of the stimulus were presented at the same time as the high saliency distractor – so a problem in stimulus-driven circuit breaking cannot be critical.

Moreover, we suggest the problem JM exhibited here not only involves disengagement but also initial attention allocation. While in Posner’s spatial disengagement a spatial cue acts to direct attention, here JM was unable to allocate attention according to a top-down cue (attend to the local or global shape) in the presence of salient distraction. Attention selection is dominated by the relative saliency of the local or global levels.

The failure to overcome bottom-up salience signals in JM also fits with recent work pointing to the PPC (and LIP in monkeys) as the locus of top-down and bottom-up interactions that yield a dynamic priority map for attention selection (see Bisley and Goldberg, 2010; Ptak, 2012 for recent reviews). More specifically, however, we have previously provided evidence both from unilateral brain lesions (Mevorach et al., 2006a) and TMS (Mevorach et al., 2006b) that the left PPC is particularly involved in ignoring salient distractors and orienting attention in a task-based manner to a low saliency target. In particular, the left PPC is involved in a preparatory selection process whereby the processing of early visual cortex signals representing salient distracters is attenuated. This attenuation process in turn facilitates selection of the less salient target. Thus, the failure in top-down selection in JM is likely to reflect an impairment in top-down attentional control modulated through the left PPC.

In sum we suggest that the deficit we observed here in JM reflects a particularly severe instance of a problem in both salience-based selection (so that selection is determined by the relative saliency of stimuli), which is typically associated with the left PPC, and in disengaging attention once the wrong (but salient) level has been selected (typically associated with the right PPC in the spatial modality). It follows that the left and right PPC damage suffered by JM may both be critical here, which results in a general problem in attentional control.

We do note, though, that JM had some problems identifying local letters even in the target salient, congruent condition. To account for this we suggest that JM had, on some trials, awareness that she had selected the wrong level of the stimulus (e.g., the global form), but the problem with attentional disengagement led to her guessing the identity of the local form.

We conclude that global processing (especially with high saliency global shapes) still operates in simultanagnosia and that an impairment in controlling attention can be a core factor that impedes the patient’s ability to actively and flexibly select the stimuli relevant to a task. This deficit impairs not only the initial selection of the stimuli but also the ability to flexibly shift attention from one level of processing to another. As a consequence selection is dominated by the relative saliency of the visual input and there is a reduced possibility that a patient can “correct” and shift selection once a salient element has been attended.

## Conflict of Interest Statement

The authors declare that the research was conducted in the absence of any commercial or financial relationships that could be construed as a potential conflict of interest.
